# Identification and Evaluation of Suitable Reference Genes for RT-qPCR Analysis in *Hippodamia variegata* (Coleoptera: Coccinellidae) Under Different Biotic and Abiotic Conditions

**DOI:** 10.3389/fphys.2021.669510

**Published:** 2021-05-17

**Authors:** Jiaoxin Xie, Tinghui Liu, Adel Khashaveh, Chaoqun Yi, Xiaoxu Liu, Yongjun Zhang

**Affiliations:** ^1^State Key Laboratory for Biology of Plant Diseases and Insect Pests, Institute of Plant Protection, Chinese Academy of Agricultural Sciences, Beijing, China; ^2^College of Plant Protections, Agricultural University of Hebei, Baoding, China

**Keywords:** RT-qPCR analysis, *Hippodamia variegata*, reference gene, expression stability, biotic conditions, abiotic conditions

## Abstract

Reverse transcriptase-quantitative polymerase chain reaction (RT-qPCR) is an accurate and convenient technique for quantifying expression levels of the target genes. Selection of the appropriate reference gene is of the vital importance for RT-qPCR analysis. *Hippodamia variegata* is one of the most important predatory natural enemies of aphids. Recently, transcriptome and genome sequencings of *H. variegata* facilitate the gene functional studies. However, there has been rare investigation on the detection of stably expressed reference genes in *H. variegata*. In the current study, by using five analytical tools (Delta Ct, geNorm, NormFinder, BestKeeper, and RefFinder), eight candidate reference genes, namely, *Actin*, *EF1*α, *RPL7*, *RPL18*, *RPS23*, *Tubulin-*α, *Tubulin-*β, and *TufA*, were evaluated under four experimental conditions including developmental stages, tissues, temperatures, and diets. As a result, a specific set of reference genes were recommended for each experimental condition. These findings will help to improve the accuracy and reliability of RT-qPCR data, and lay a foundation for further exploration on the gene function of *H. variegata*.

## Introduction

The reverse transcriptase-quantitative polymerase chain reaction (RT-qPCR) is one of the most sensitive, accurate, and convenient methods for detecting quantitative analysis of gene expression in biological samples (Kubista et al., 2006; Provenzano and Mocellin, 2007; Derveaux et al., 2010). For RT-qPCR studies, it is necessary to select appropriate reference genes to correct and standardize the expression level of target genes. This will reduce the impact of RNA quality and cDNA synthesis efficiency on the results. Therefore, the validity of candidate internal reference gene needs to be evaluated before conducting RT-qPCR assay.

Reference genes, also called housekeeping genes, are supposed to be stably expressed in organisms under various biotic or abiotic conditions (Thellin et al., 1999; de Jonge et al., 2007; Zhu et al., 2014). The ideal internal reference gene should be stably expressed in different types of tissues and in different treatments of the same tissue, and its expression level is not affected by any endogenous or exogenous factors (Brym et al., 2013; Janská et al., 2013). Nevertheless more and more researches proved that the internal reference genes show significant various expression levels in different experimental treatments such as development stages, tissues, cells, and temperatures (Sun et al., 2008; Huis et al., 2010). Therefore, it is necessary to identify reference genes for their expression under different experimental conditions (Guo et al., 2016; Wan et al., 2017; Renard et al., 2018). Common reference genes include *actin*, *glyceraldehyde-3-phosphate dehydrogenase* (*GAPDH*), *ribosomal protein S3* (*RPS3*), *18S ribosomal RNA* (*18S*), *25S ribosomal RNA* (*25S*), *tubulin*, *translation elongation factor 1-alpha* (*EF1*α), and others have been used extensively for RT-qPCR analysis (Lu et al., 2013, 2015; Yuan et al., 2014; Zhang et al., 2015; Ma et al., 2016; Yan et al., 2016; Gao et al., 2017; Hu et al., 2018; Yin et al., 2020).

*Hippodamia variegata* (Coccinellidae: Hippodamia) is one of the most important natural enemies of aphids infesting various crops in many countries (Natskova, 1973; Hameed et al., 1975; Belikova and Kosaev, 1985). The stably expressed reference genes in coccinellid insects such as *Harmonia axyridis* (Yang et al., 2018), *Henosepilachna vigintioctomaculata* (Lü et al., 2018a), *Coccinella septempunctata* (Yang et al., 2016), and *Hippodamia convergens* (Pan et al., 2015) have been screened under different experimental conditions. In this study, eight reference genes for insects, including *actin*, *elongation factor-1*α (*EF1*α), *ribosomal protein L7* (*RPL7*), *ribosomal protein L18* (*RPL18*), *ribosomal protein L23* (*RPL23*), *tubulin alpha* (*Tubulin-*α), *tubulin beta* (*Tubulin-*β), and *elongation factor Tu* (*TufA*), have been selected as candidate reference genes for *H. variegata*. The expression stabilities of these genes were assessed with respect to different developmental stages, tissues, temperatures, and diet treatments using five different statistical algorithms. The five statistical algorithms are Delta Ct method (Silver et al., 2006), geNorm (Vandesompele et al., 2002), NormFinder (Andersen et al., 2004), BestKeeper (Pfaffl et al., 2004), and RefFinder (Xie et al., 2012), which integrates four different statistical algorithms. Finally, the expression pattern of *olfactory co-receptor* (*Orco*) of *H. variegata* was assayed to verify the reference genes. The results will serve as a guide for gene expression studies in *H. variegata*.

## Materials and Methods

### Insect Rearing

The *H. variegata* were obtained from Langfang Experimental Station of Chinese Academy of Agricultural Sciences, Hebei Province, China. Larvae and adults were maintained in plastic containers (8 × 8 × 11 cm) with *Myzus persicae* as a food source. The colonies were reared under conditions of 25 ± 1°C, 65 ± 10% relative humidity, and a 14:10-h light/dark photoperiod.

### Sample Treatment and Collection

Samples at developmental stages including eggs (50 eggs), first-instar larvae (10 individuals), second-instar larvae (10 individuals), third-instar larvae (2 individuals), fourth-instar larvae (1 individual), pupae (1 individual), and adults (1 male and 1 female individual) were selected as different treatment groups. Moreover, different tissues including head, thorax, abdomen, leg, and wing were dissected and harvested from adult females or males at 2 to 3 days (*N* = 30).

To evaluate the effects of temperature, third-instar larvae of *H. variegata* were exposed to 15, 25, and 35°C for 5 h. In diet treatment groups, 23-instar larvae of *H. variegata* were fed 3-day-old larvae of *Spodoptera frugiperda* (FAW), nymphs and adults of *M. persicae*, and canola pollen separately after 12 h of starvation (Schuldiner-Harpaz and Coll, 2017a, b).

Each experiment was replicated three times independently. Samples were preserved in 1.5-ml centrifuge tubes and snap frozen immediately in liquid nitrogen before storage at –80°C.

### Total RNA Extraction and Reverse Transcription

Total RNA was extracted using TRIzol (Invitrogen, Carlsbad, CA, United States) following the manufacturer’s instructions. RNA purity was checked on a NanoDrop spectrophotometer (Thermo Fisher Scientific, Waltham, MA, United States). Each sample of 2.0 μg total RNA was used to synthesize single-stranded cDNA using the FastQuant RT kit (Tiangen Biotech, Beijing Co., Ltd., Beijing, China).

### Selection of Candidate Reference Genes

The reciprocal BLAST hits approach was used to screen reference genes from *H. variegata* transcriptome and genome data (unpublished data). The reference genes from other insect species in GenBank were downloaded from NCBI^1^ queried individually to *H. variegata* transcriptome and genome using the TBLASTN program with a permissive *E*-value cutoff of 10^–5^ to get the hits. And then, each of the queried hits was compared back against non-redundant database of NCBI by the BLASTX program (*E* < 10^–5^) to determine whether the original sequence was one of the hits. All the selected reference genes are listed in Table 1.

**TABLE 1:** Primers used for candidate reference genes.

**Genes**	**Accession no.**	**Primer sequences (5′–3′)**	**Length (bp)**	**Efficiency (%)**	***R*^2^**	**Linear regression**
*Actin*	MT721831	F: CGAAAGCAGAAGAGCATAG	157	87.98	0.9851	y = –3.6483x + 21.669
		R: TCAGTTAGAAGCACAGGAT				
*EF1*α	MT721832	F: AGCCAACATTACCACTGA	127	98.67	0.9995	y = –3.3541x + 15.535
		R: GTATCCACGACGCAATTC				
*RPL7*	MT721833	F: GGATATGCGAACCCTACATA	110	92.63	0.9963	y = –3.5122x + 16.941
		R: GGTGATTGGTATTCTCTGTC				
*RPL18*	MT721834	F: TGACCATTTGTGCTTTGAA	150	96.49	0.997	y = –3.409x + 12.282
		R: ATTCTCTGGCGTTCCTAC				
*RPS23*	MT721835	F: CCGTTGTGGTTTGATGTAT	198	103.42	1	y = –3.2427x + 21.289
		R: AATGAACTTCTGTGTTAAGGTT				
*Tubulin-*α	MT721836	F: GCATTGGTATGTTGGTGAA	123	103.34	0.9936	y = –3.2444x + 26.011
		R: GCTTCATTGTCAGTATCATCA				
*Tubulin-*β	MT721837	F: GTGGCTGTTTGTGATGTT	103	92.1	0.9984	y = –3.5269x + 19.33
		R: ACTGTTCGTGGATTCTTCT				
*TufA*	MT721838	F: TGCTGGATAGTATTGATGATTAC	112	91.24	0.9996	y = –3.5514x + 21.434
		R: ACCACAACAGTTCCTCTT				

The primers of eight candidate reference genes were designed using Beacon Designer TM 7.9 software (Premier Biosoft International, CA, United States) according to sequences obtained from our recently sequenced transcriptomes and genome for *H. variegata*. Primer parameters were as follows: optimal temperature 60 ± 2°C, GC content 40–60%, and 18–24 bp in length. The amplification size was between 100 and 200 bp (Table 1).

Gene-specific primers (Supplementary Table 1) were designed using Primer Premier 6.0 software (Premier Biosoft International, CA, United States) to amplify ORF of *Orco* gene. PCR amplifications were performed in 50-μl reactions containing 25 μl 2 × Phanta Max Master Mix, 2 μl of each primer (10 μM each), 4 μl first-strand cDNA, and 17 μl ddH_2_O. The PCR parameters were as follows: one cycle of 94°C for 3 min; 35 cycles of 94°C for 30 s, 55°C for 30 s, and 72°C for 1 min; a final cycle of 72°C for 10 min. PCR products were purified and cloned into the pcloneEZ-Blunt TOPO vector (Clone Smarter, United States) for sequencing confirmation.

### RT-qPCR Analysis

The RT-qPCR measurements were performed on an ABI Prism 7500 system (Applied Biosystems, Carlsbad, CA, United States). The reaction system contained a mixture of 12.5 μl 2 × SuperReal PreMix Plus (Tiangen Biotech, Beijing Co., Ltd.), 0.75 μl of each primer (10 μM), 1 μg sample cDNA, 0.6 μl 50 × ROX Reference Dye, and proper volume of RNase-free water. PCR cycling parameters were as follows: 95°C for 15 min, followed by 40 cycles of 95°C for 10 s, and cooled to 60°C for 32 s. Then, the PCR products were heated to 95°C for 15 s, cooled to 60°C for 1 min, heated to 95°C for 30 s, and cooled to 60°C for 15 s to measure the dissociation curves. To determine the amplification efficiency (E) and the correlation coefficients (R^2^), fivefold dilution series of cDNA (1:5, 1:25, 1:125, 1:625, and 1:3,125) were used as templates to construct a standard curve of each gene. Also, the homologous RT-qPCR efficiencies (E) were calculated according to the equation: *E* = (10^[–1/slope]^ - 1) × 100.

### Analysis of the Stability of Reference Gene Expression

Five tools (Delta Ct, geNorm, NormFinder, BestKeeper, RefFinder) were used to evaluate the stability of each reference gene. The Delta Ct method was used to select the optimal reference gene (Silver et al., 2006). geNorm was used to calculate the normalization factor to determine the optimal number of reference genes (Vandesompele et al., 2002); the proposed Vn/Vn + 1 ratio below *V* ≤ 0.15 does not significantly affect the normalization. The NormFinder algorithm, a model-based method, was used to rank the candidate reference genes by the stability value, and the gene with the lowest value was considered as the most stable reference gene (Andersen et al., 2004). The BestKeeper was used to rank the candidate reference genes based on the SDs and the coefficients of variation (CV) values (Pfaffl et al., 2004). Finally, RefFinder assigned an appropriate weight of the four methods to a single gene and calculated the geometric average of their weights for the overall final ranking (Xie et al., 2012).

### Validation of the Selected Reference Genes

The *Orco* is highly conserved among insect species and is essential for localization of ORs in olfactory sensory neuron (ORN) dendrites and reception of odor signals (Leal, 2013). Thus, *Orco* was used as the target gene to assess the stability of candidate reference genes. Based on the selected candidate reference gene set, *Orco* expression levels in different tissues were calculated. The primers are shown in Supplementary Table 1. The gene expression was measured in various tissues and normalized by the optimal reference genes (*TufA* and *RPS23*) and the least stable reference genes (*Tubulin-*α and *Tubulin-*β). RT-qPCR analysis was performed as described previously. Data were calculated using the 2^–△△Ct^ method (Schmittgen and Livak, 2008) and the expression level of target gene (*Orco*) has been normalized with the *CT*-value average of two best and least stable reference genes. All the experiments were performed in three replications, and the results are expressed as means ± SD.

## Results

### RT-qPCR Analysis

**FIGURE 1:**
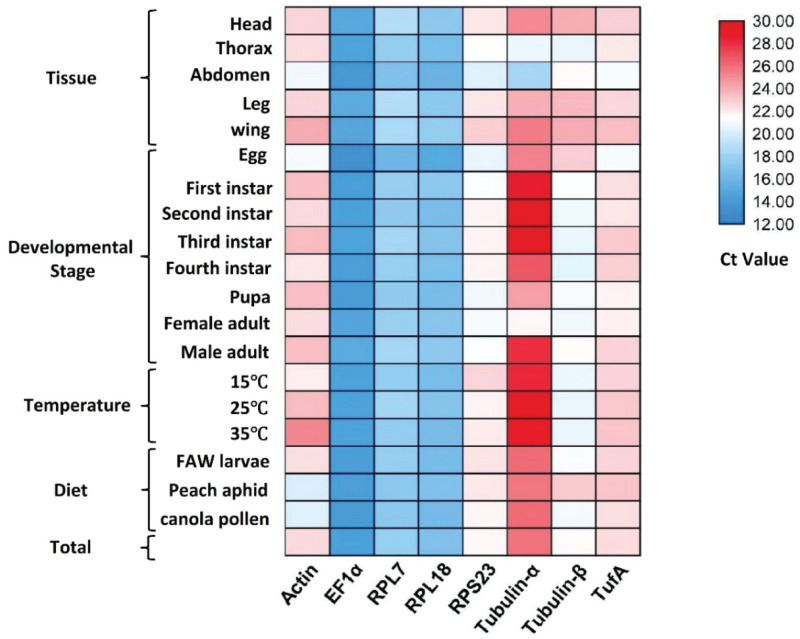
*Ct*-value of eight candidate reference genes under different experimental conditions. Numbers represent *Ct*-values.

Before evaluating the applicability of the reference genes, the specificity and efficiency of PCR amplification should be first ascertained. PCR amplifications for each primer pair showed a single peak in melt curves (Supplementary Figure 1). The primer efficiency (E) ranged between 87.98 and 103.42% with linear regression coefficient (R^2^) values of 0.9851–1 (Table 1). Combining the three aforementioned factors, the results showed that the designed primers can precisely amplify candidate reference genes. *Ct*-values of the eight candidate reference genes ranged from 13.2 to 29.6, covering all the experimental conditions (Figure 1). *EF1*α was the most abundant reference gene with the lowest *Ct*-value, followed by *RPL18*, *RPL7*, *Tubulin-*β, *RPS23*, *TufA*, *Actin*, and *Tubulin-*α.

### Expression Stability of Candidate Reference Genes Under Biotic Conditions

**FIGURE 2:**
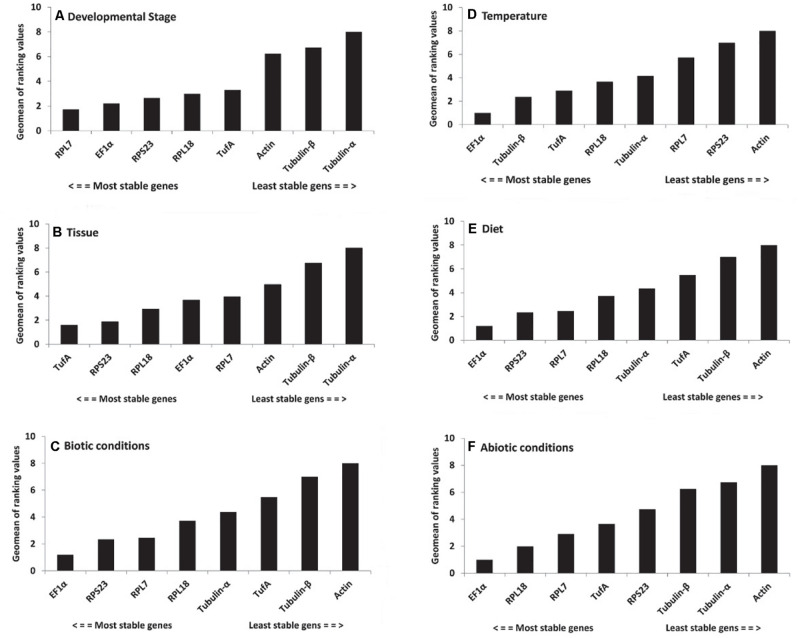
Expression stability of candidate reference genes under biotic and abiotic experimental conditions. **(A)** Development stages, **(B)** tissues, **(C)** biotic conditions, **(D)** temperatures, **(E)** diets, and **(F)** abiotic conditions. A lower Geomean value suggests stable expression.

**FIGURE 3:**
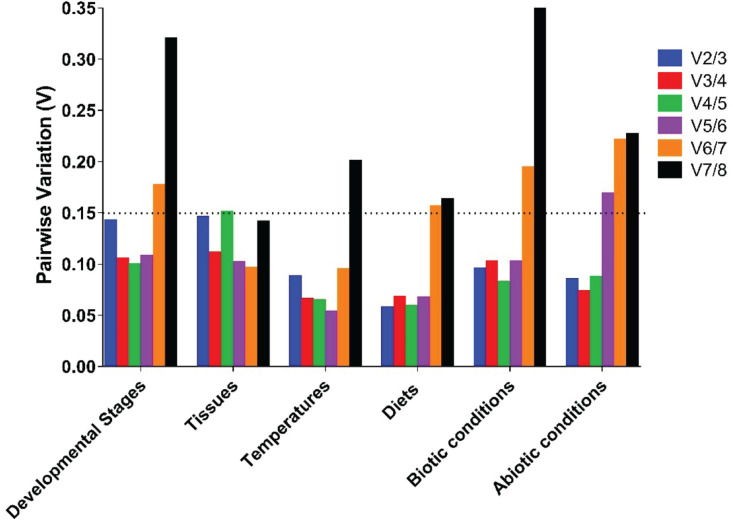
Optimal number of reference genes required for accurate normalization of gene expression by geNorm. Based on geNorm analysis, average pairwise variations were calculated between the normalization factors NFn and NFn + 1. Values <0.15 indicate that n + 1 genes were not required for the normalization of gene expression.

For different developmental stages, geNorm ranked the stability from high to low as *RPL7* = *RPL18*, *EF1*α, *TufA*, *RPS23*, *Actin*, *Tubulin-*β, and *Tubulin-*α. NormFinder provided a ranking as *RPS23*, *TufA*, *RPL7*, *EF1*α, *RPL18*, *Actin*, *Tubulin-*β, and *Tubulin-*α. BestKeeper offered a list as follows: *EF1*α, *RPS23*, *RPL7*, *RPL18*, *Tubulin-*β, *TufA*, *Actin*, and *Tubulin-*α. Delta Ct provided a ranking as *RPL7*, *EF1*α, *TufA*, *RPL18*, *RPS23*, *Actin*, *Tubulin-*β, and *Tubulin-*α. Combining the results of all four programs, RefFinder identified the two candidates *RPL7* and *EF1*α with the highest consensus at different developmental stages. *RPL7* was the most stable gene, whereas *Tubulin-*α was the most unstable candidate (Figure 2A and Table 2). The geNorm results demonstrated that the initial *V*-value at V2/3 < 0.015, indicating that only two reference genes were required to normalize the target gene data (Figure 3).

**TABLE 2:** Expression stability of eight candidate reference genes in *Hippodamia variegata* under different experimental conditions using five programs.

**Condition**	**Reference genes**	**geNorm**		**NormFinder**		**BestKeeper**		**Delta Ct**		**RefFinder**		**Recommendation**
		**Stability**	**Rank**	**Stability**	**Rank**	**SD**	**Rank**	**Stability**	**Rank**	**Genes Geomean (GM)**	**Rank**	
Developmental stages												*RPL7*, *EF1*α
	*Actin*	0.499	6	0.543	6	0.70	7	1.03	6	6.24	6	
	*EF1*α	0.285	3	0.362	4	0.37	1	0.88	2	2.21	2	
	*RPL7*	0.231	1	0.338	3	0.47	3	0.85	1	1.73	1	
	*RPL18*	0.231	1	0.426	5	0.49	4	0.90	4	2.99	4	
	*RPS23*	0.429	5	0.118	1	0.38	2	0.93	5	2.66	3	
	*Tubulin-*α	1.210	8	2.534	8	2.13	8	2.60	8	8.00	8	
	*Tubulin-*β	0.747	7	1.357	7	0.55	5	1.59	7	6.74	7	
	*TufA*	0.376	4	0.185	2	0.55	5	0.89	3	3.31	5	
Tissues												*TufA*, *RPS23*
	*Actin*	0.423	6	0.487	5	0.70	4	0.88	5	4.95	6	
	*EF1*α	0.361	5	0.732	6	0.42	1	0.91	6	3.66	4	
	*RPL7*	0.309	4	0.353	3	0.73	5	0.80	4	3.94	5	
	*RPL18*	0.217	3	0.361	4	0.59	2	0.73	3	2.91	3	
	*RPS23*	0.155	1	0.077	1	0.77	6	0.71	2	1.86	2	
	*Tubulin-*α	1.029	8	2.250	8	2.60	8	2.29	8	8.00	8	
	*Tubulin-*β	0.608	7	0.819	7	1.30	7	1.20	7	7.00	7	
	*TufA*	0.155	1	0.143	2	0.68	3	0.70	1	1.57	1	
Temperatures												*EF1*α, *Tubulin-*β
	*Actin*	0.711	8	1.590	8	1.08	8	1.61	8	8.00	8	
	*EF1*α	0.11	1	0.071	1	0.04	1	0.49	1	1.00	1	
	*RPL7*	0.285	5	0.334	6	0.31	5	0.59	6	5.73	6	
	*RPL18*	0.211	3	0.260	5	0.21	4	0.53	3	3.66	4	
	*RPS23*	0.411	7	0.765	7	0.37	7	0.86	7	7.00	7	
	*Tubulin-*α	0.309	6	0.104	2	0.31	5	0.57	5	4.16	5	
	*Tubulin-*β	0.11	1	0.224	4	0.06	2	0.53	3	2.38	2	
	*TufA*	0.249	4	0.104	2	0.17	3	0.50	2	2.91	3	
Diets												*EF1*α, *RPS23*
	*Actin*	0.731	8	1.285	8	0.98	8	1.33	8	8.00	8	
	*EF1*α	0.135	1	0.067	2	0.11	1	0.49	1	1.19	1	
	*RPL7*	0.165	3	0.044	1	0.19	4	0.54	3	2.45	3	
	*RPL18*	0.221	4	0.092	4	0.15	2	0.54	3	3.72	4	
	*RPS23*	0.135	1	0.067	2	0.21	5	0.50	2	2.34	2	
	*Tubulin-*α	0.259	5	0.370	6	0.15	2	0.63	6	4.36	5	
	*Tubulin-*β	0.535	7	1.190	7	0.88	7	1.23	7	7.00	7	
	*TufA*	0.309	6	0.223	5	0.30	6	0.6	5	5.48	6	
Biotic conditions												*RPL18*, *RPL7*
	*Actin*	0.492	6	0.372	5	0.69	6	1.13	6	5.73	6	
	*EF1*α	0.303	3	0.461	6	0.43	1	1.01	5	3.08	5	
	*RPL7*	0.277	1	0.336	4	0.57	3	0.97	3	2.45	2	
	*RPL18*	0.277	1	0.292	3	0.54	2	0.96	1	1.57	1	
	*RPS23*	0.408	5	0.169	1	0.57	3	1	4	2.99	3	
	*Tubulin-*α	1.381	8	3.232	8	2.58	8	3.28	8	8.00	8	
	*Tubulin-*β	0.748	7	1.467	7	1.13	7	1.73	7	7.00	7	
	*TufA*	0.371	4	0.169	1	0.60	5	0.96	1	2.99	3	
Abiotic conditions												*EF1*α, *RPL18*
	*Actin*	1.114	8	1.783	8	1.46	8	1.88	8	8.00	8	
	*EF1*α	0.188	1	0.094	1	0.12	1	0.76	1	1.00	1	
	*RPL7*	0.242	3	0.130	2	0.25	3	0.82	4	2.91	3	
	*RPL18*	0.188	1	0.213	4	0.18	2	0.79	2	2.00	2	
	*RPS23*	0.351	5	0.485	5	0.26	4	0.91	5	4.73	5	
	*Tubulin-*α	0.859	7	1.276	6	1.37	7	1.50	7	6.74	7	
	*Tubulin-*β	0.577	6	1.353	7	0.64	6	1.45	6	6.24	6	
	*TufA*	0.278	4	0.131	3	0.28	5	0.80	3	3.66	4	

For the tissue-specific experiment, geNorm, NormFinder, and Delta Ct identified *TufA* and *RPS23* as the most suitable reference genes, and BestKeeper identified *EF1*α and *RPL18* as the most suitable reference genes (Table 2). Through the calculation of RefFinder, the overall ranking of the eight candidate internal reference genes was as follows: *TufA* > *RPS23* > *RPL18* > *EF1*α > *RPL7* > *Actin* > *Tubulin-*β > *Tubulin-*α (Figure 2B and Table 2). The geNorm pairwise variation analysis also revealed that the first *V* < 0.15 emerged at V2/3 (Figure 3). Thus, the best combination of reference genes for tissue-specific samples of *H. variegata* was *TufA* and *RPS23*.

According to the comprehensive ranking of RefFinder, under biological conditions, the most stable to the least stable candidate reference genes were *RPL18*, *RPL7*, *TufA*, *RPS23*, *EF1*α, *Actin*, *Tubulin-*β, and *Tubulin-*α (Figure 2C and Table 2). The geNorm results identified an initial *V* < 0.015 at V2/3, indicating that only two references genes were needed to normalize the target gene data (Figure 3). Thus, the best combination of reference genes for tissue-specific samples of *H. variegata* was *RPL18* and *RPL7*.

### Stability of Candidate Reference Genes Under Abiotic Conditions

Under different temperature conditions, combining the results of four software analysis, the RefFinder rankings were as follows: *EF1*α, *Tubulin-*β, *TufA*, *RPL18*, *Tubulin-*α, *RPL7*, *RPS23*, and *Actin* (Figure 2D and Table 2). Through geNorm pairwise variation analysis, the results showed that the two reference genes were sufficient to normalize the target genes in different temperatures (Figure 3). Therefore, for *H. variegata* at different temperatures, the best combination of reference genes is *EF1*α and *Tubulin-*β.

For the diet treatment, geNorm and Delta Ct identified *EF1*α and *RPS23* as the most suitable reference genes, NormFinder identified *EF1*α and *RPL7*, whereas BestKeeper identified *EF1*α and *RPL18* as the most suitable reference genes (Table 2). Based on RefFinder analysis, the comprehensive reference genes are ranked from the most stable to the most unstable as follows: *EF1*α, *RPS23*, *RPL7*, *RPL18*, *Tubulin-*α, *TufA*, *Tubulin-*β, and *Actin* (Figure 2E and Table 2). In addition, geNorm pairwise variation analysis indicated that two reference genes were sufficient to normalize the target gene in dietary treatments (Figure 3). Therefore, we suggest that the best combination of reference genes in dietary treatments is *EF1*α and *RPS23*.

According to comprehensive ranking of RefFinder, under abiotic conditions, the order of the most stable to unstable candidate reference genes was as follows: *EF1*α, *RPL18*, *RPL7*, *TufA*, *RPS23*, *Tubulin-*β, *Tubulin-*α, and *Actin* (Figure 2F and Table 2). The geNorm results identified an initial *V* < 0.015 at V2/3, indicating that only two references genes were required to standardize the target gene data (Figure 3). Therefore, under different abiotic status, the best combination of reference genes of *H. variegata* was *EF1*α and *RPL18*.

### Validation of Selected Reference Genes

**FIGURE 4:**
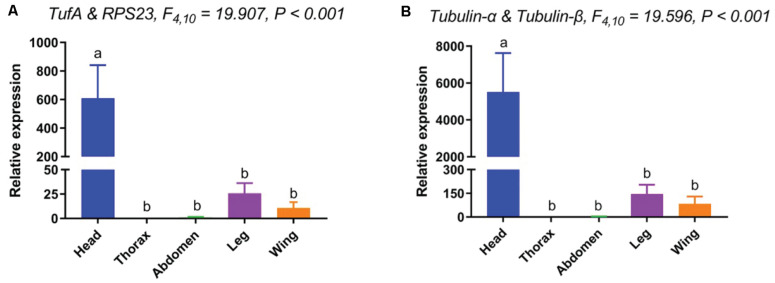
Relative expression levels of *Orco* in different tissues. The relative mRNA expression levels of *Orco* were normalized to the most suited [**(A)**. *TufA* and *RPL23*) and the least suited [**(B)**. *Tubulin-*α and *Tubulin-*β) reference genes. Values are means ± SEM. Different letters indicate significant differences (*P* < 0.05, two-way ANOVA followed by Tukey’s HSD multiple comparison).

When standardized with the two most stable reference genes (*TufA* and *RPL23*), the expression pattern of *Orco* in various tissues were similar. The expression level of *Orco* was as follows: head > leg > wing > abdomen > thorax. However, the expression level of *Orco* in the head was almost 600-fold higher than in the leg when the two most stable reference genes were used for normalization (Figure 4A). In contrast, when the two most unstable reference genes (*Tubulin-*α and *Tubulin-*β) were used for normalization, the *Orco* expression in the head was tremendously high. The calculated relative expression of *Orco* in head with two unstable genes was almost 10 times higher than that of normalized with two stable genes (Figure 4B).

## Discussion

*H. variegata* is one of the most important predatory natural enemies of aphids in the Xinjiang Uygur Autonomous Region, the main cotton-producing area of China, which shows a strong control effect on cotton aphids (Feng et al., 2000). The genome and transcriptomes of both sexes of *H. variegata* have been sequenced recently (unpublished data). To further investigate the biological roles of any specific gene in *H. variegata*, quantitative gene expression through RT-qPCR is essential. Following the “Minimum Information for Publication of Quantitative Real-Time PCR Experiments” (MIQE) guideline, reference genes with high stability are necessary (Bustin et al., 2009). However, to our knowledge, the best or combination of best reference genes under various treatment conditions have not been identified in *H. variegata* so far.

In this study, BestKeeper (Pfaffl et al., 2004), geNorm (Vandesompele et al., 2002), NormFinder (Andersen et al., 2004), and Delta values (Silver et al., 2006) were used to evaluate the expression stability of the candidate internal reference genes. The overall stability of selected reference genes were also evaluated using RefFinder (Xie et al., 2012), which is a web-based analysis tool that integrates all four major calculation programs. Because of the different algorithms, the stability levels derived from the four analysis tools may be different. Optimal number of reference genes could be determined using geNorm analysis by calculating the paired mutation value (Vn/n + 1). When Vn/n + 1 is <0.15, there is no need to add additional reference genes to improve accuracy (Vandesompele et al., 2002).

The expression stability of eight candidate reference genes showed that the most suitable candidate combinations of reference genes were as follows: *RPL7* and *EF1*α for developmental stages, *TufA* and *RPS23* for tissues, *EF1*α and *Tubulin-*β for temperature treatment, *EF1*α and *RPS23* for diet treatment, *RPL18* and *RPL7* for biotic conditions, and *EF1*α and *RPL18* for abiotic conditions. The *EF1*α, encoding a protein associated with translation elongation, is the most abundant protein in the cell and highly conserved in different species (Yin et al., 2020). Several research works have been reported that *EF1*α is the most stable reference gene in different group of insect species such as *Lymantria dispar* (Lepidoptera: Lymantriidae) (Yin et al., 2020), *Cydia pommnella* (Lepidoptera: Tortricidae) (Wei et al., 2020), *Aphis gossypii* (Hemiptera: Aphididae) (Ma et al., 2016), *Locusta migratoria manilensis* (Orthoptera: Acrididae) (Yang et al., 2014), and *H. convergens* (Pan et al., 2015). The ICG website^2^ counts the top 10 reported internal reference genes, among which, *EF1*α is in the first rank (Sang et al., 2017).

The *RPL18* encodes a ribosomal protein that is a component of the 60S subunit and belongs to the L18E family of ribosomal proteins (Fagerberg et al., 2014). It has been demonstrated that under different experimental conditions, *RPL18* is also one of the most stable reference genes in different group of insects such as *Solenopsis invicta* (Hymenoptera, Formicidae) (Cheng et al., 2013), *Anastrepha obliqua* (Diptera, Tephritidae) (Nakamura et al., 2016), and *Cimex lectularius* (Hemiptera, Cimicidae) (Mamidala et al., 2011). Consistently, *RPL18* is stably expressed under different biotic and abiotic conditions and could be considered as a proper reference gene for normalization of RT-qPCR data in *H. variegata*. Similar to *EF1*α, RPL family genes are also ranked among the top five stable reference genes for RT-qPCR analysis by ICG website (Sang et al., 2017).

Ribosomal protein S family including *RPS23* has also been reported as the most appropriate reference gene in insects (Fu et al., 2013) and other organisms (Yadav et al., 2012) and has been ranked seventh by the ICG website. In contrast, *TufA* is not a commonly used reference gene in insects (Lü et al., 2018b). However, based on our preliminary evaluation and main experimental data, it has been shown that *TufA* was stably expressed in different tissues and could be considered as a reference gene in *H. variegata*. Previous studies demonstrated that in different experimental conditions, *TufA* is one of the most stable reference genes in different species of bacteria (Savard and Roy, 2009; Jiang et al., 2019).

The *Orco* is highly conserved in all insects and is widely expressed in the most olfactory receptor neurons (ORNs) of olfactory sensilla, distributed on the head (mainly expressed in antennae and mouthparts), legs, wings, and abdomen (Jacquin-Joly and Merlin, 2004). To validate our obtained results, the two most stable reference genes and the two most unstable reference genes were applied for normalization of *Orco*. Using the two most stable internal reference genes to examine the expression pattern of *Orco*, the results were stable and almost similar in different tissues. On the other hand, by using the two most unstable internal reference genes, the relative expression level of *Orco* normalized with *Tubulin-*β was approximately double lower in the head than when normalized with *Tubulin-*α. A similar phenomenon was also observed when *OBP20* was used as the target gene to verify the stability of the internal reference gene of *Apolygus lucorum* (Hemiptera, Miridae). The relative expression level of *OBP20*, normalized with the stable reference genes (*RPL32* and *RPL27*), was approximately threefold higher in the head than when normalized with two unstable reference genes (*ACT* and β*ACT*) (Luo et al., 2019). Under two normalization conditions, the expression of target genes in same tissue (head) was different, indicating that the inappropriate use of reference genes in any experimental condition may lead to inaccurate results. Therefore, selection and validation of the best reference genes are crucial to determine the accuracy of the expression pattern of different genes. Taken together, our findings provide a more rigorous method to normalize RT-qPCR data in *H. variegata*, which lays a foundation for the functional genomics research in the future.

## Data Availability Statement

The original contributions presented in the study are included in the article/Supplementary Material, further inquiries can be directed to the corresponding author/s.

## Author Contributions

JX and AK conceived and designed the research. JX and CY conducted the experiments. TL and XL analyzed the data. JX and YZ wrote the article. All authors have read and agreed to the published version of the article.

## Conflict of Interest

The authors declare that the research was conducted in the absence of any commercial or financial relationships that could be construed as a potential conflict of interest.

## Footnotes

1 http://www.ncbi.nlm.nih.gov/

2 http://icg.big.ac.cn
