# Supplementary material for: Identification and Evaluation of Suitable Reference Genes for RT-qPCR Analysis in Hippodamia variegata (Coleoptera: Coccinellidae) Under Different Biotic and Abiotic Conditions
Source: Front Physiol. 2021 May 17;12:669510. doi: 10.3389/fphys.2021.669510 (PMC8165390; doi:10.3389/fphys.2021.669510)
Supplement: Supplementary file 1 [file Data_Sheet_1.docx]

**Supplementary:**

Figure S1. Melt Curves of the eight candidate reference genes.
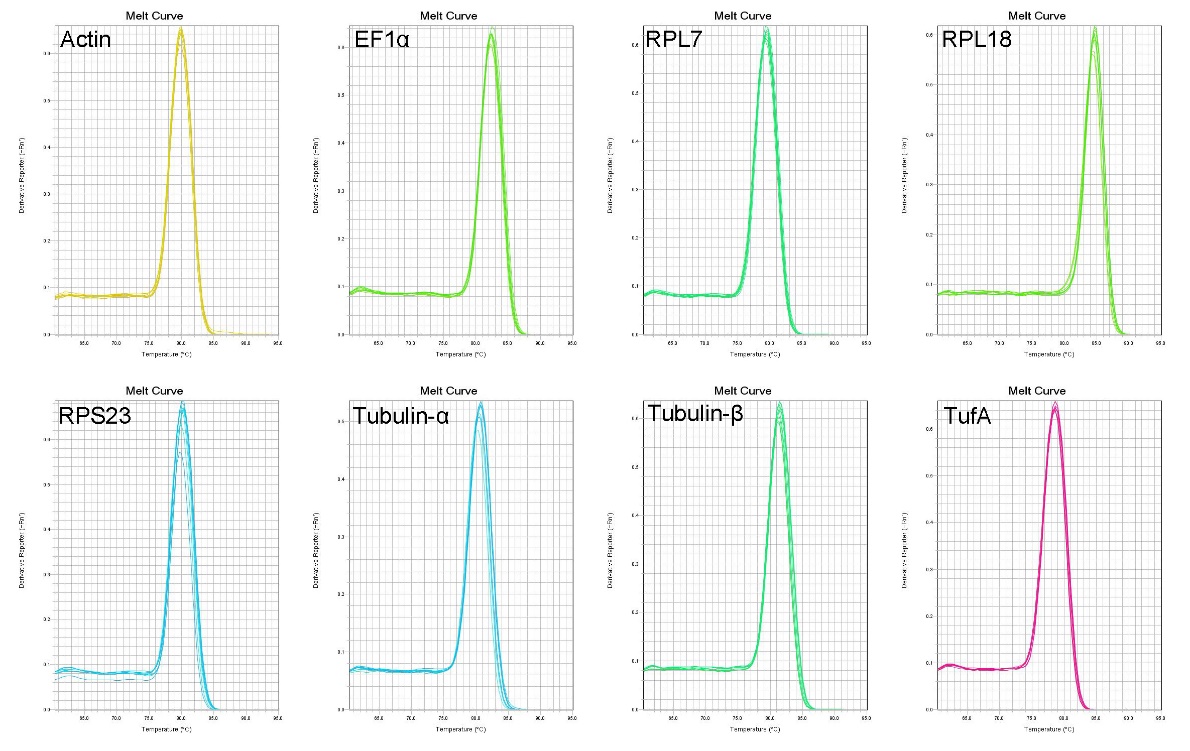


Table S1. Primer sequences of eight candidate genes for clone and *Orco* for RT-qPCR.

| Primer name | Sequence (5’-3’） |
| --- | --- |
| ***Gene clone*** |  |
| Actin-forward | ATGAAAATGGAAACCTATGATGTC |
| Actin-reverse | TTAGAACATTTTTCGATGGACAG |
| EF1α-forward | ATGGGTAAAGAAAAGACTCATATTAAC |
| EF1α-reverse | CTACTTCTTCTTTGTTGCTTTTTCG |
| RPL7-forward | ATGGCGCCAACTGTAGAAAAATC |
| RPL7-reverse | TTAAACCATCCTTCTGAGGAGTTC |
| RPL18-forward | ATGGGTATAGATATCGAACACAAATATG |
| RPL18-reverse | CTACTTCTTGTAACCACATGACTTCC |
| RPS23-forward | ATGGCTGCTAGTAGATTAGAAAAAATC |
| RPS23-reverse | TCAATTGAAAACTTTACTAAGGTCAA |
| Tubulin-α-forward | ATGTCCGACATAATTTCACTTCA |
| Tubulin-α-reverse | TCATTGCTCCATGGACAATG |
| Tubulin-β-forward | ATGAGGGAAATAATCCAAATTCAAG |
| Tubulin-β-reverse | TTAACAACAGTGACTGCTGTTAATG |
| TufA-forward | ATGACATTCGACTTTGACGTAACA |
| TufA-reverse | TTAATCAGGTAACACTAATTTTCCTA |
| ***RT-qPCR*** |  |
| Orco-forward | CAGAACACTCGGTATATGGA |
| Orco-reverse | TTAATCGTATGGTTGCTTGG |
